# Molecular characterization of m6A RNA methylation regulators with features of immune dysregulation in IgA nephropathy

**DOI:** 10.1007/s10238-024-01346-8

**Published:** 2024-05-02

**Authors:** Yihao Wang, Nan Sun, Rui He, Zida Wang, Jingsi Jin, Ting Gao, Junwen Qu

**Affiliations:** 1 Department of Urology, Jiading Branch, Renji Hospital, Shanghai Jiao Tong University School of Medicine, Shanghai, 201899 China; 2 Department of Nephrology, Shanghai Changzheng Hospital, Naval Medical University, Shanghai, 200003 China; 3 Department of Urology, Renji Hospital, Shanghai Jiao Tong University School of Medicine, Shanghai, 200127 China; 4 Shanghai Immune Therapy Institute, Renji Hospital, Shanghai Jiao Tong University School of Medicine, Shanghai, 200127 China; 5 Department of Emergency, Renji Hospital, Shanghai Jiao Tong University School of Medicine, Shanghai, 200127 China

**Keywords:** IgA nephropathy, RNA methylation, Immune infiltrates, m6A, Nomogram

## Abstract

The role of RNA N6-methyladenosine (m6A) modification in immunity is being elucidated. This study aimed to explore the potential association between m6A regulators and the immune microenvironment in IgA nephropathy (IgAN). The expression profiles of 24 m6A regulators in 107 IgAN patients were obtained from the Gene Expression Omnibus (GEO) database. The least absolute shrinkage and selection operator (LASSO) regression and logistic regression analysis were utilized to construct a model for distinguishing IgAN from control samples. Based on the expression levels of m6A regulators, unsupervised clustering was used to identify m6A-induced molecular clusters in IgAN. Gene set enrichment analysis (GSEA) and immunocyte infiltration among different clusters were examined. The gene modules with the highest correlation for each of the three clusters were identified by weighted gene co-expression network analysis (WGCNA). A model containing 10 m6A regulators was developed using LASSO and logistic regression analyses. Three molecular clusters were determined using consensus clustering of 24 m6A regulators. A decrease in the expression level of YTHDF2 in IgAN samples was significantly negatively correlated with an increase in resting natural killer (NK) cell infiltration and was positively correlated with the abundance of M2 macrophage infiltration. The risk scores calculated by the nomogram were significantly higher for cluster-3, and the expression levels of m6A regulators in this cluster were generally low. Immunocyte infiltration and pathway enrichment results for cluster-3 differed significantly from those for the other two clusters. Finally, the expression of YTHDF2 was significantly decreased in IgAN based on immunohistochemical staining. This study demonstrated that m6A methylation regulators play a significant role in the regulation of the immune microenvironment in IgAN. Based on m6A regulator expression patterns, IgAN can be classified into multiple subtypes, which might provide additional insights into novel therapeutic methods for IgAN.

**Supplementary Information:**

The online version contains supplementary material available at 10.1007/s10238-024-01346-8.

## Introduction

IgA nephropathy (IgAN) is the most common form of glomerulonephritis and is caused by the deposition of IgA immunoglobulin in the glomerular basement membrane (GBM). Immune-mediated damage to the GBM results in hematuria, proteinuria, and renal insufficiency [1]. IgAN can be classified into primary and secondary forms, and secondary IgAN can be caused by Henoch-Schönlein purpura, lupus, inflammatory bowel disease, and hepatitis [2]. A systematic review of biopsy-based studies conducted primarily in European countries suggests an overall population incidence of at least 2.5 adults per 100,000 population diagnosed with IgAN [3]. The true incidence is likely even higher, particularly in Asian and Pacific Asian countries, as some patients with IgAN display no obvious symptoms and therefore do not undergo kidney biopsy [4]. The deposition of IgA subclass poly IgA1, which contains a J-chain, in the mesangial region serves as the initiating event in the pathogenesis of IgAN. The codeposition of autoantibodies against galactose-deficient IgA1, IgG, and complement C3 antibodies is also common and may be related to disease severity. Antibody deposition in the mesangial region and the activation of complement factors result in mesangial hypercellularity, endocapillary hypercellularity, segmental glomerulosclerosis, and atrophying interstitial fibrosis [5, 6]. Studies have found that the O-galactosylation of IgA1 is also regulated by microRNA (miRNA), indicating that IgA galactose deficiency may be regulated posttranscriptionally [7]. Recently, RNA posttranscriptional modifications have emerged as another epigenetic regulatory mechanism for gene expression. Therefore, understanding the RNA regulatory mechanisms associated with IgAN might be helpful for understanding the underlying pathologic mechanisms.

Almost 110 different modified bases have been identified in RNA across various RNA types, such as messenger RNA (mRNA), transfer RNA (tRNA), ribosomal RNA (rRNA), and others [8]. These modifications not only mark and regulate these transcripts but also diversify and extend the functions of proteins generated by these annotated genes. Among all identified modifications, N6-methyladenosine (m6A) is the most common internal modification made to mRNAs and long noncoding RNAs (lncRNAs). The m6A methylation modification is a highly dynamic and reversible process that involves methyltransferases (writers), demethylases (erasers), and binding proteins (readers) [9]. Members in each class of regulators work together in a coordinated manner to maintain a steady-state balance of m6A levels within the cell. The m6A modification plays an important role in the immune process, and an imbalance in m6A modifications and regulatory factors has been associated with various diseases, particularly different types of cancers [10]. Therefore, we speculated that m6A regulators might play a potential role in the pathogenesis of IgAN, specifically in the immune response to galactose-deficient IgA1. By studying immune differences between IgAN and other types of glomerulonephritis in addition to changes in the expression of m6A regulators across IgAN subtypes, we can enhance our understanding of the pathogenesis of IgAN from a new perspective.

In this study, we systematically analyzed the expression levels of 24 m6A regulators in IgAN. Differences in the abundance of immunocytes among different types of nephritis were examined to explore the immune characteristics of IgAN. We observed a correlation between immunocyte abundance and the expression levels of m6A regulators. IgAN patients were classified into three clusters using an unsupervised clustering approach. Different immune traits were associated with each cluster, and the biological functions of each cluster were examined through enrichment analysis. Finally, the gene modules with the highest correlation for each subtype were obtained by weighted gene co-expression network analysis (WGCNA), which will lay the foundation for our subsequent research.

## Methods

### Data collection and preprocessing

The microarray expression profile for IgAN was obtained from the Gene Expression Omnibus (GEO) database (https://www.ncbi.nlm.nih.gov/geo). The search strategy was based on combinations of key words: “IgA nephropathy”, “IgA glomerulonephritis”, “IgA nephrosis”, “IgAN” and “IgA kidney disease” without language restrictions. We got a total of 37 datasets. The inclusion criteria for data selection were as follows: (1) the sample was glomerular tissue, (2) the study type was limited to expression profiling by microarray, (3) the organism was restricted to *Homo sapiens*, and (4) the sample size was greater than 50. The mRNA expression matrices for GSE115857 and GSE116626 were selected for inclusion in our analysis. Analyses of both datasets were performed on the GPL14951 platform (Illumina HumanHT-12 WG-DASL V4.0 R2 expression bead chip). Microarray batch effects were removed using the “ComBat” function in the R package “sva” (version 3.8) from the Bioconductor project (http://www.bioconductor.org/). After the initial normalization step, the data from both gene expression arrays were combined for all subsequent analyses.

### Analysis of m6A RNA regulators in IgAN and control samples

The expression levels of 24 m6A regulators in the gene expression arrays were extracted and analyzed. The expression relationships among these 24 m6A regulators were evaluated using a Spearman’s correlation analysis across all samples. The expression level differences for all 24 m6A regulators between the IgAN and control samples were compared using the Wilcoxon test.

### The development of a nomogram to identify IgAN

Least absolute shrinkage and selection operator (LASSO) regression was used to screen for variables that differentiate between IgAN and control samples. LASSO regression is a shrinkage method that can select from a large and potentially multicollinear set of variables, resulting in a more relevant and interpretable set of predictors [11]. We utilized the package “glmnet” (version 4.1-2) to perform the LASSO regression. A both-ward, stepwise, logistic regression based on multivariate analysis was then used to develop a model, which was expressed in the form of a nomogram. We plotted a calibration curve to estimate the calibration of the nomogram. Receiver operating characteristic (ROC) curve analysis was used to quantify the discrimination performance of the nomogram.

### Identification of m6A-induced molecular clusters of IgAN

Unsupervised clustering analysis was performed using the R package “ConsensusClusterPlus” (version 2.7) to classify IgAN patients into different clusters based on the expression of 24 m6A regulatory factors. The clustering was performed using 100 iterations, with each iteration containing 80% of the samples. Consensus clustering was based on resampling to verify the rationality of clustering, and its main purpose was to evaluate the robustness of the clustering analysis. The optimal cluster number was determined using cumulative distribution function (CDF) curves of the consensus score and the clear separation of the consensus matrix heatmaps.

### Analysis of biological functions and immune traits among different clusters

Gene set enrichment analysis (GSEA) is a computational method that assesses whether a set of a priori defined genes shows significant, concordant differences between different subtypes [12]. The GSEA hallmark pathway was used to identify predefined gene sets; 1000 permutations were performed according to the gene set to determine adjusted *p* values. A pathway with an adjusted *p* value < 0.05 and a false discovery rate (FDR) < 0.25 was considered to be significant.

The CIBERSORT algorithm is an online method for estimating the abundances of member cell types in a mixed cell population of complex tissues based on gene expression profiles (https://cibersort.stanford.edu/) [13]. Using the existing “LM22” signature matrix file available on the CIBERSORT website, we calculated the infiltration level of 22 immunocytes based on the RNA expression profile data for all samples. After permutation testing with 1000 replicates, a deconvolution *p* < 0.05 was recognized as significant. Immunocyte abundances were compared between IgAN and control samples using the Wilcoxon test. The same approach was used to compare immunocyte abundances among the IgAN subtypes. The correlation between m6A regulators and immunocyte fractions was determined with a Spearman’s correlation analysis.

### Identification of m6A-related gene modules

WGCNA constructs molecular networks to excavate hidden information by grouping genes with a high degree of co-expression into the same modules, taking advantage of differences in gene expression [14]. The core steps of WGCNA network construction are as follows. (1) A gene expression similarity matrix was constructed by calculating the Pearson’s correlation coefficients for all gene pairs. (2) An adjacency matrix was established according to the soft threshold β value transformations. (3) WGCNA applied a topological overlap matrix (TOM) to calculate the degree of association between genes. (4) Modules were identified using hierarchical gene clustering with dynamic tree cutting, using 1 − TOM as the distance measurement and a minimum module size cutoff of 50 genes. (5) Highly similar modules were clustered and merged together using a height cutoff of 0.3, allowing for the most significant modules to be identified.

### Renal tissues and immunohistochemistry

Renal tissues from IgAN patients (n = 4) were collected as a portion of a biopsy specimen acquired for diagnostic pathology. Control kidney samples (n = 4) were obtained as a portion of a zero-time biopsy specimen of donor kidneys. IgAN patients and control individuals were from Renji Hospital of Shanghai Jiao Tong University, Shanghai, China. Immunohistochemical staining was carried out by incubation of the tissue sections with YTHDF2 and IGF2BP1antibody (Proteintech Group, Inc., Chicago, IL, USA) and visualization of color development with diaminobenzidine.

## Results

### Heterogeneity of m6A regulators between IgAN and control samples

**Fig. 1:**
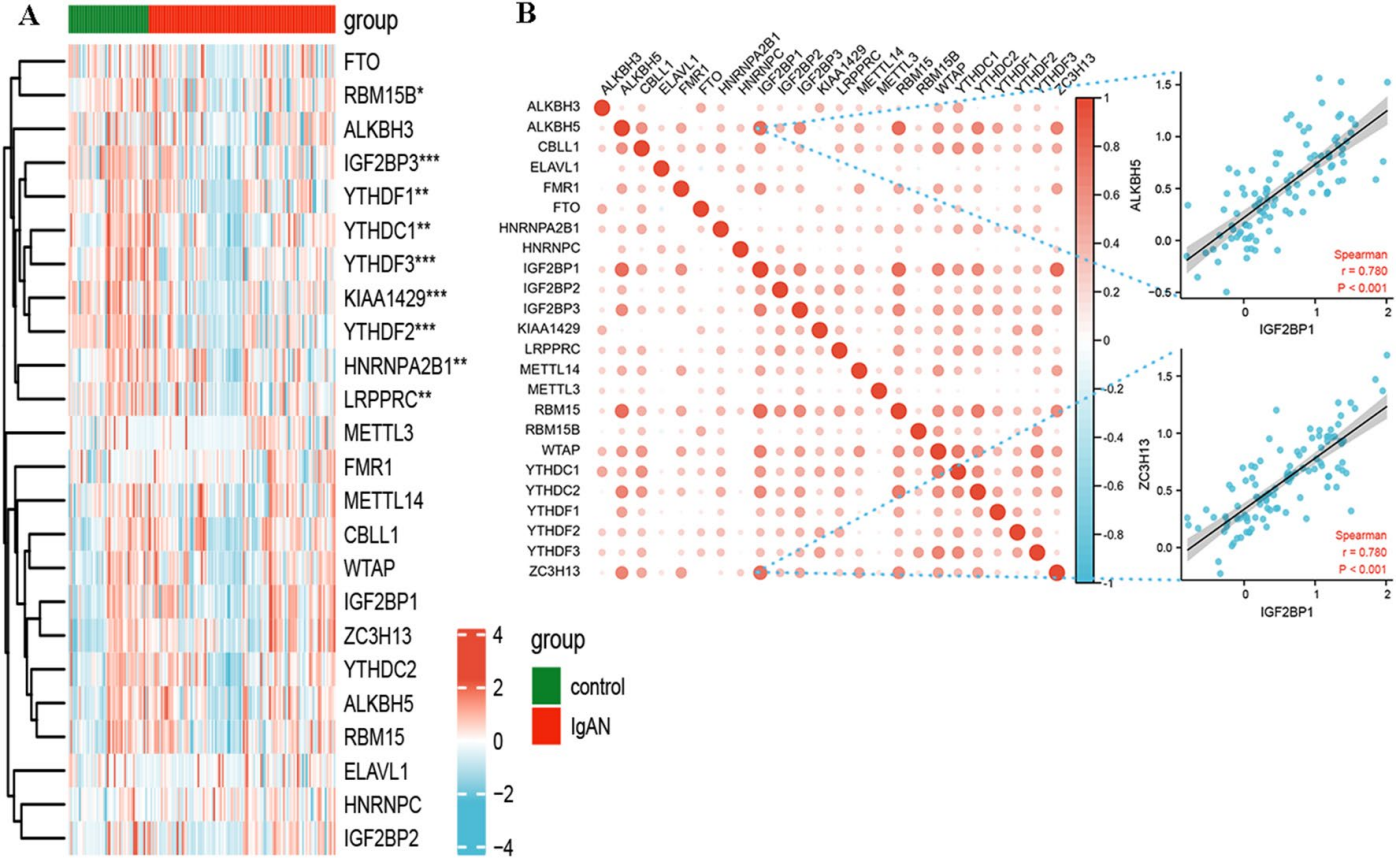
Expression landscape of m6A RNA regulators in IgAN. (A) The heatmap plot demonstrated the expression status of 24 m6A regulators between IgAN and healthy samples. (B) Correlations among the expression of 24 m6A regulators in all samples. The two scatter-plots demonstrated that IGF2BP1 was significantly correlated with ALKBH5 and ZC3H13. *p < 0.05; **p < 0.01; ***p < 0.001

The GSE115857 dataset, which includes data from renal biopsy tissues, contained 55 IgAN samples and 24 control samples. The GSE116626 dataset contained 52 IgAN samples and 22 control samples. After removing batch effects from the microarray data, the two gene matrices were merged into a new dataset for subsequent analysis. A total of 24 m6A regulators were examined in this study, including 8 writers, 13 readers, and 3 erasers (Table 1). A differential expression analysis between IgAN and control samples identified significant differences in the expression levels of 9 m6A regulators, including YTHDF2, IGF2BP3, KIAA1429, YTHDF1, YTHDC1, YTHDF3, HNRNPA2B1, LRPPRC and RBM15B, which were reduced in IgAN samples compared with those of control samples; YTHDF2 presented the most significant difference (Fig. 1A). Correlation analysis of the m6A regulators in all samples indicated that IGF2BP1 was significantly correlated with the expression of multiple m6A regulators, particularly ALKBH5 and ZC3H13, suggesting that IGF2BP1 may have a more significant effect than other m6A regulators (Fig. 1B).

**Table 1:** 24 m6A RNA methylation regulators were collected in this study

Gene symbol	Type
METTL3	M6A writers
METTL14	M6A writers
WTAP	M6A writers
KIAA1429	M6A writers
RBM15	M6A writers
RBM15B	M6A writers
CBLL1	M6A writers
ZC3H13	M6A writers
YTHDF1	M6A readers
YTHDF2	M6A readers
YTHDF3	M6A readers
YTHDC1	M6A readers
YTHDC2	M6A readers
HNRNPC	M6A readers
HNRNPA2B1	M6A readers
IGF2BP1	M6A readers
IGF2BP2	M6A readers
IGF2BP3	M6A readers
FMR1	M6A readers
ELAVL1	M6A readers
LRPPRC	M6A readers
FTO	M6A erasers
ALKBH5	M6A erasers
ALKBH3	M6A erasers

### The development of a nomogram to distinguish IgAN

**Fig. 2:**
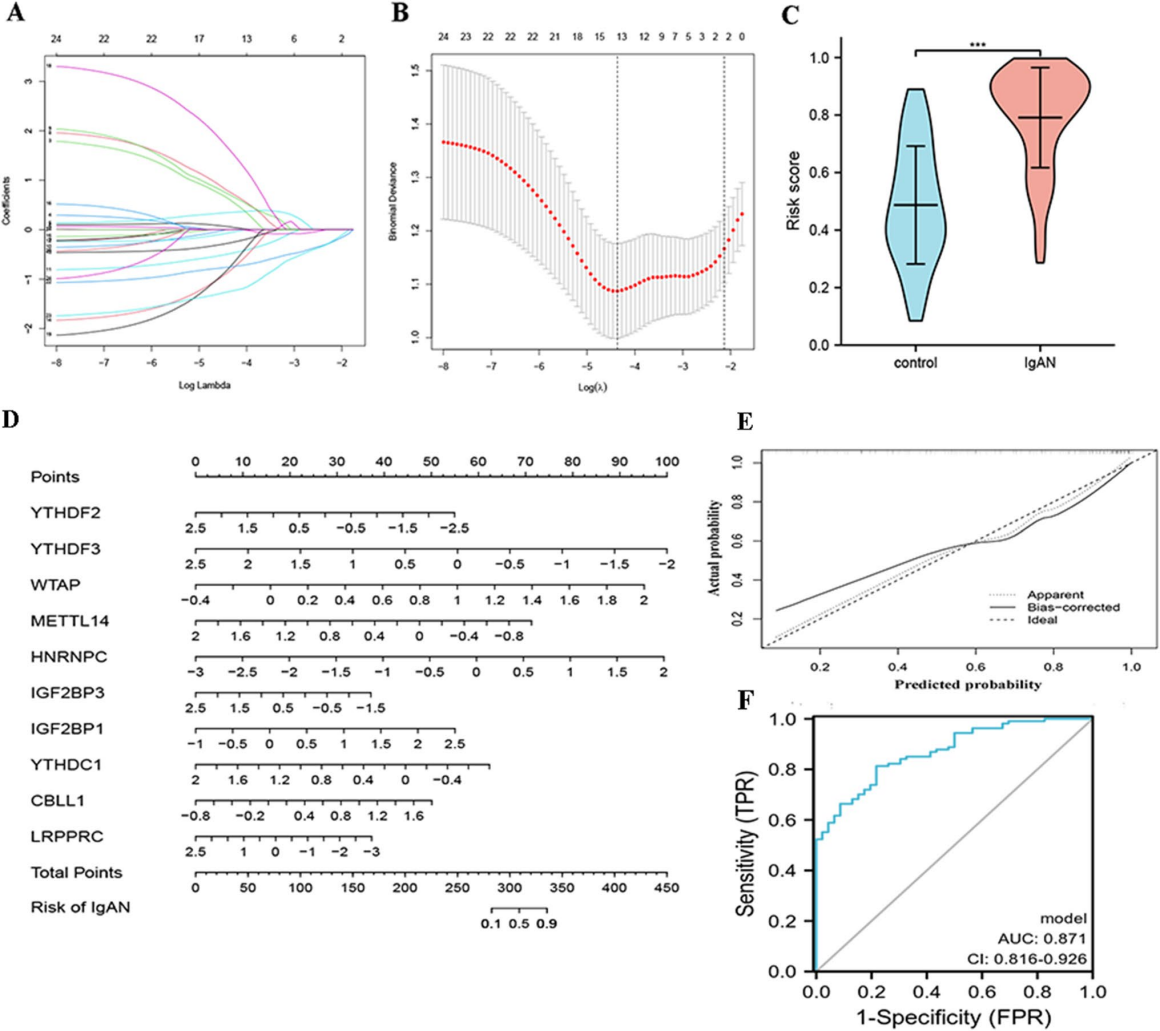
The m6A regulators can distinguish healthy samples and IgAN. **A** Least absolute shrinkage and selection operator (LASSO) coefficient profiles of 24 IgAN-related m6A regulators. **B** tenfold cross-validation for tuning parameter selection in the LASSO regression. The partial likelihood deviance is plotted against log (λ), where λ is the tuning parameter. Partial likelihood deviance values are shown, with error bars representing SE. The dotted vertical lines are drawn at the optimal values by minimum criteria and 1-SE criteria. **C** The risk distribution between IgAN and healthy samples, where IgAN have a much higher risk score than healthy samples. **D** Example of a nomogram for distinguishing IgAN and healthy samples. (To use the nomogram, an individual score is located on each variable axis, and a line is drawn upward to determine the scores from each variable value. The sum of these scores is located on the Total Points axis, and a line is drawn downward to determine the risk of IgAN). **E** The calibration curve for determining the risk of IgAN. **F** The discrimination ability for healthy and IgAN samples by m6A regulators was analyzed by ROC curve and evaluates by AUC value

LASSO regression was performed on all 24 m6A regulators as a preliminary screening of variables that may be related to the incidence of IgAN (Fig. 2A, B). Based on the minimum standard, 13 m6A regulators were selected to participate in model construction (Table 2), and the risk scores calculated by LASSO regression for IgAN patients were significantly higher than those calculated for control samples (Fig. 2C). Subsequently, multivariate logistic regression was performed to develop a model capable of distinguishing IgAN from control samples. The novel model for IgAN identification, which contained 10 m6A regulators, including CBLL1, HNRNPC, IGF2BP1, IGF2BP3, LRPPRC, METTL14, WTAP, YTHDC1, YTHDF2, YTHDF3, is presented in the form of a nomogram (Fig. 2D). A calibration plot for the probability of IgAN identification showed agreement between the nomogram prediction and actual observations (Fig. 2E). The Hosmer–Lemeshow test for the nomogram showed that Pearson’s χ^2^ value was 5.751 with a *p* value of 0.675. The ROC curve was used to calculate the area under the curve (AUC) for the ability of the nomogram to predict IgAN and resulted in an AUC of 0.871 (95% confidence interval [CI]: 0.816–0.926; Fig. 2F). These results suggest that the nomogram had good discrimination and calibration for IgAN.

**Table 2:** Multivariate logistic regression of 13 m6A RNA methylation regulators

Variable	*β*	Odds ratio (95% CI)	*p*
ALKBH3	0.197	1.218 (0.768–1.931)	0.403
CBLL1	1.594	4.923 (0.966–25.089)	0.055
FMR1	0.229	1.257 (0.499–3.168)	0.627
HNRNPC	1.513	4.54 (0.957–21.542)	0.057
IGF2BP1	1.384	3.991 (1.129–14.108)	0.032
IGF2BP3	− 0.752	0.471 (0.221–1.005)	0.051
LRPPRC	− 0.539	0.584 (0.284–1.199)	0.143
METTL14	− 1.918	0.147 (0.04–0.533)	0.004
RBM15B	− 0.299	0.742 (0.369–1.49)	0.401
WTAP	3.137	23.042 (2.042–260.044)	0.011
YTHDC1	− 2.257	0.105 (0.013–0.819)	0.032
YTHDF2	− 0.925	0.396 (0.2–0.787)	0.008
YTHDF3	− 1.571	0.208 (0.054–0.796)	0.022

### Identification of m6A-induced molecular IgAN subtypes

**Fig. 3:**
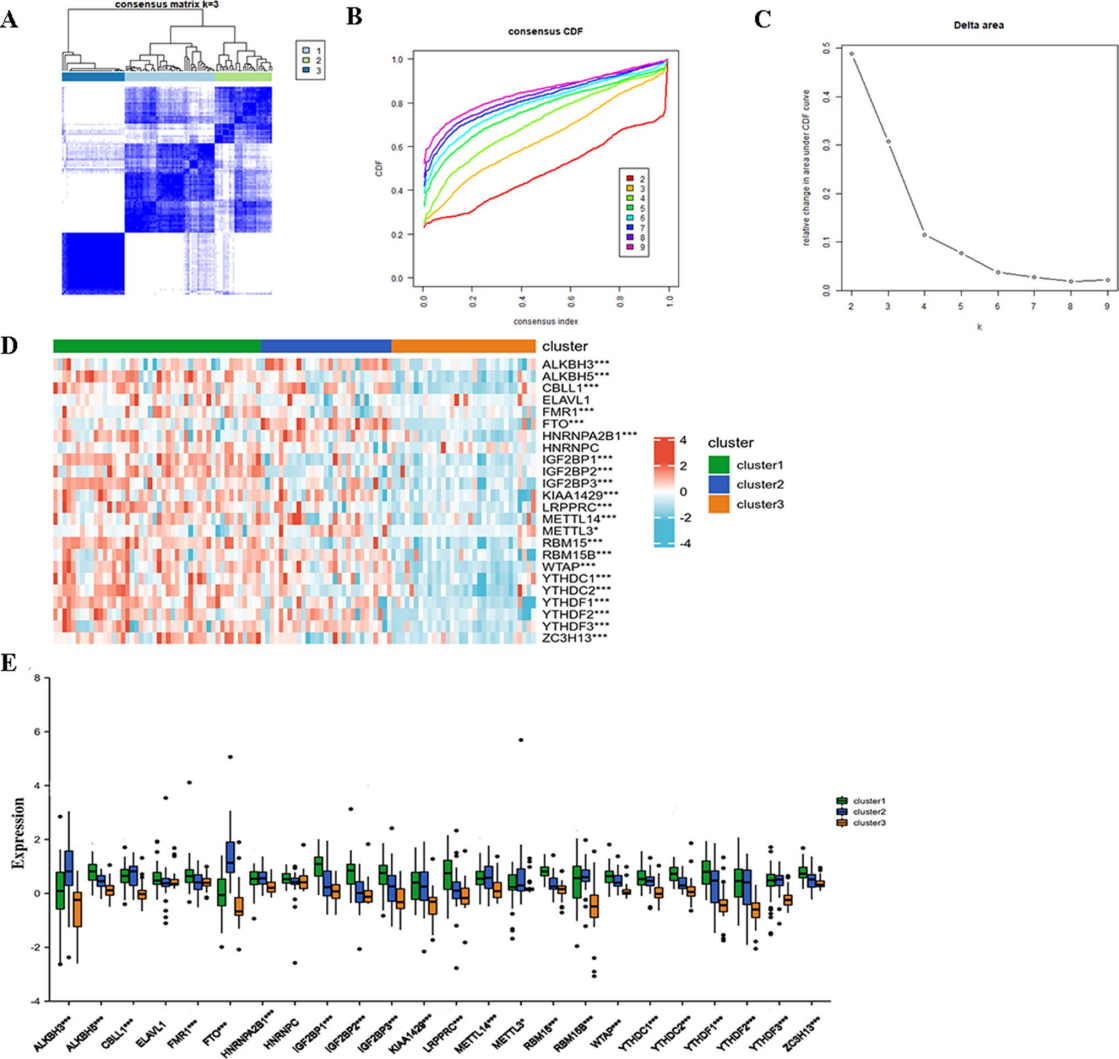
Unsupervised clustering of 24 m6A regulators. Identifying 3 distinct m6A modification pattern subtypes in IgAN. **A** Heatmap of the matrix of co-occurrence proportions for IgAN samples. **B** Consensus clustering cumulative distribution function (CDF) for k = 2–9. **C** Relative change in area under CDF curve for k = 2–9. **D** Unsupervised clustering of 24 m6A regulators in the 3 modification patterns. **E** The expression status of 24 m6A regulators in the 3 m6A subtypes. *p < 0.05; **p < 0.01; ***p < 0.001

Based on the similarity between expression patterns for the 24 m6A regulators, we performed an unsupervised consensus cluster analysis of IgAN samples. Three distinct IgAN clusters were identified based on CDF curves of the consensus score and showed clear separation on the consensus matrix heatmaps (Fig. 3A–C). Cluster-1 contained 46 samples, cluster-2 contained 29 samples, and cluster-3 contained 32 samples. Significant differences in the expression of almost all 24 m6A regulators were observed among the three clusters, confirming the diversity of m6A modification clusters in IgAN (Fig. 3D). From the box diagram, the expression levels of m6A regulators in cluster-3 were generally lower than those in the other two clusters (Fig. 3E), suggesting differences in the m6A modification patterns among different IgAN clusters.

### Analysis of biological functions and immune traits among subtypes

**Fig. 4:**
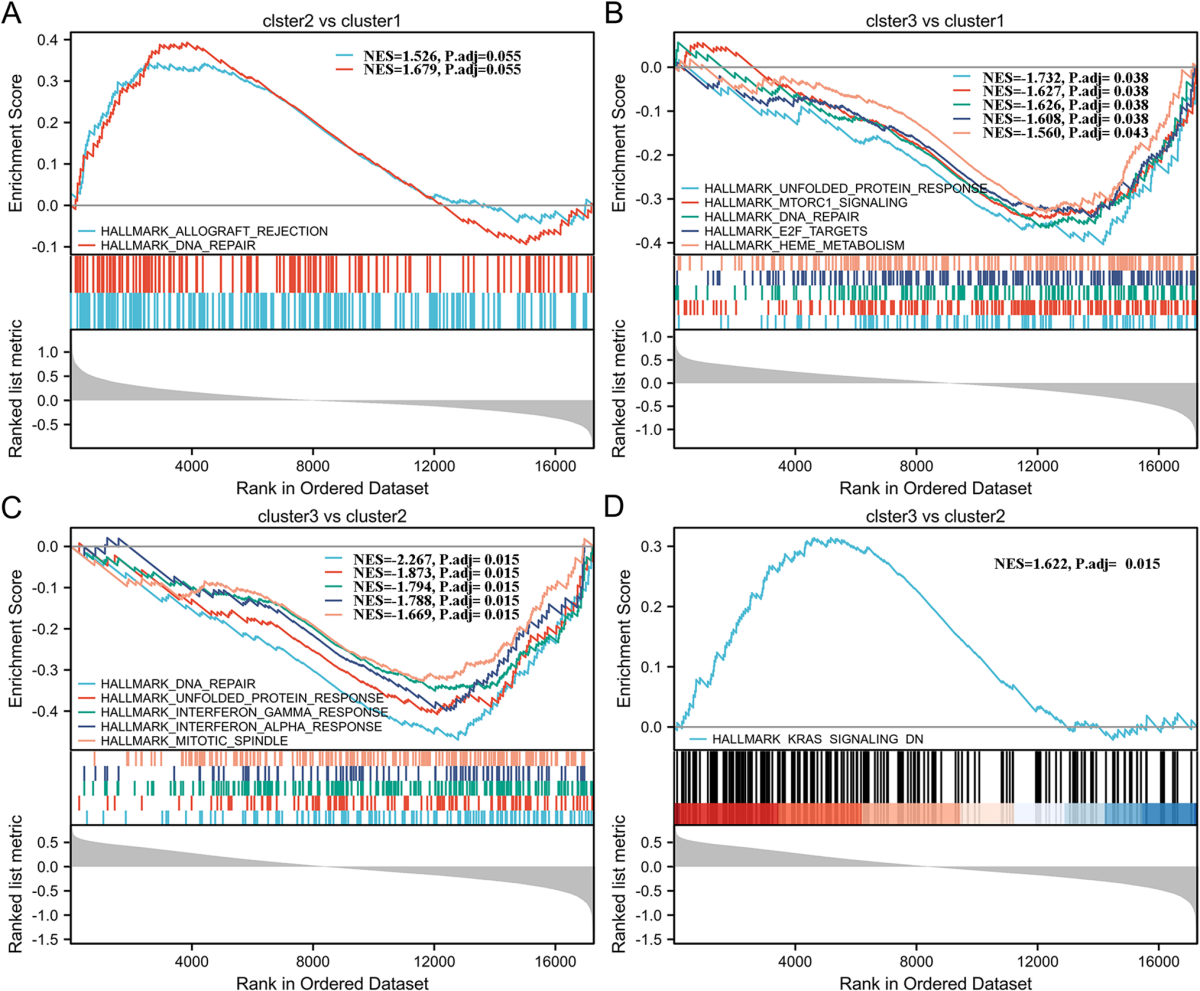
GSEA enrichment plot shows the difference of signaling pathways involved between each cluster. **A** There is no obvious difference of signaling pathways involved between cluster-1 and cluster-2. GSEA shows the top 5 downregulated signaling pathways involved between cluster-3 and cluster-1 (**B**), between cluster-3 and cluster-2 (**C**). **D** GSEA shows the upregulated signaling pathways involved between cluster-3 and cluster-2

We compared the GSEA hallmark pathway among clusters to evaluate the activation status of biological pathways. No significant differences in the activation status of hallmark pathways were observed between cluster-1 and cluster-2 (adjusted *p* < 0.05, FDR < 0.25, Fig. 4A). Comparing cluster-1 and cluster-3, a total of 5 gene sets were enriched, including the unfolded protein response, DNA repair, and cell cycle pathways (Fig. 4B). When comparing hallmark pathways between cluster-2 and cluster-3, a total of 14 gene sets were enriched. The top 5 downregulated gene sets, based on the normalized enrichment score (NES), were involved in DNA repair, the unfolded protein response, and the interferon response (Fig. 4C). Uniquely, the KRAS signal was upregulated in cluster-3 (Fig. 4D).

**Fig. 5:**
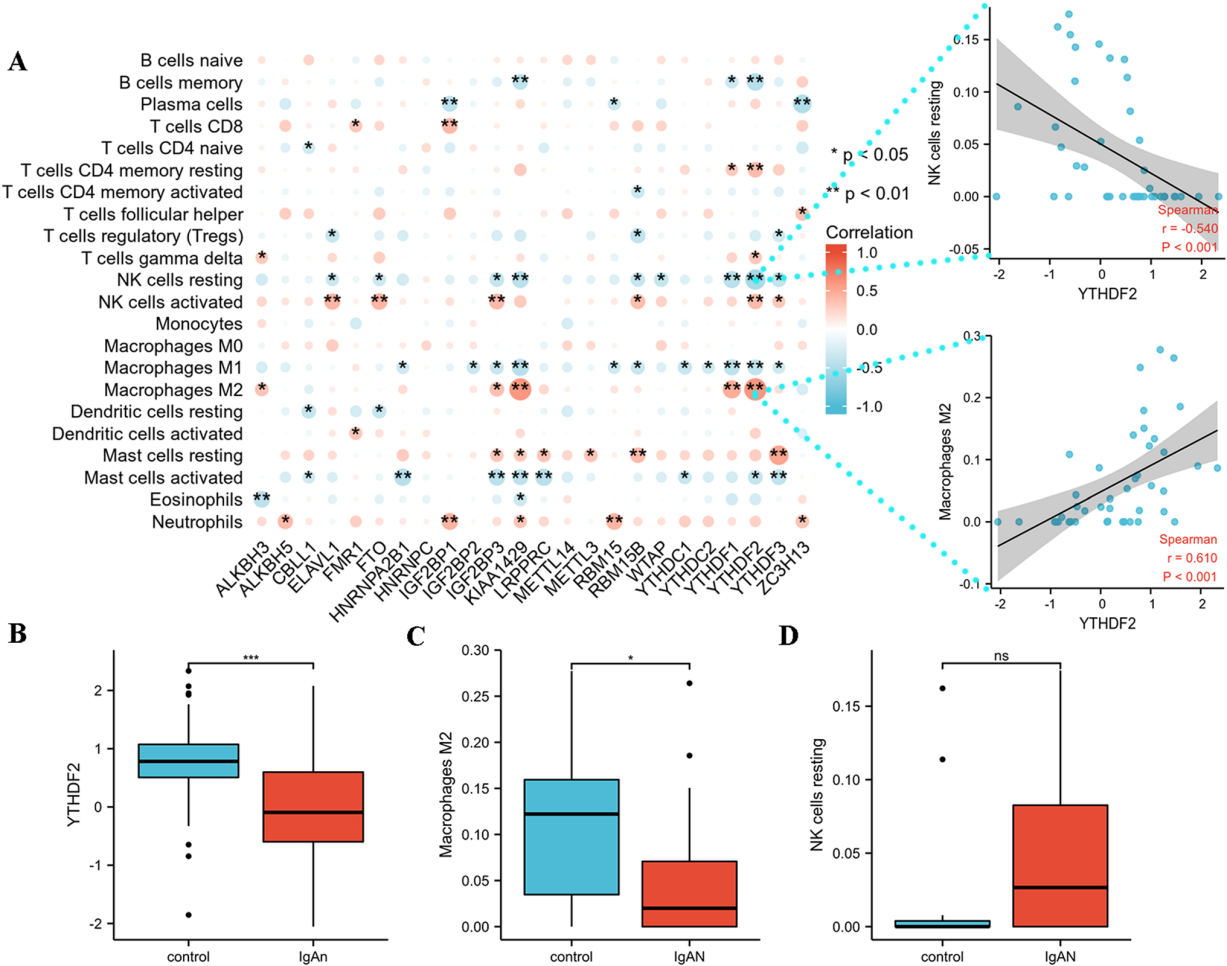
The correlation between infiltrating immunocytes and m6A regulators. **A** The dot-plot demonstrated the correlations between each immune microenvironment infiltration cell type and m6A regulator. The most positive correlated immunocyte-m6A regulator pair is YTHDF2-macrophrages M2, and the most negative correlated immunocyte-m6A regulator pair is YTHDF2-NK cell resting. **B** The expression status is presented by box plot, indicating the lower expression of YTHDF2 in IgAN. **C** The expression status is presented by box plot, indicating the lower expression of macrophrages M2 in IgAN. **D** The expression status is presented by box plot, indicating that there is no difference of expression of NK cell resting between two group. *p < 0.05; **p < 0.01; ***p < 0.001

Using the CIBERSORT online tool, we obtained 22 immunocyte infiltration fractions for each sample. A total of 43 samples were included in the immune infiltration analysis, including 11 in the control group and 32 in the IgAN group, using a *p* < 0.05 as the cutoff criterion. Correlation analysis identified that the m6A regulator YTHDF2 was significantly correlated with various immunocytes, particularly resting natural killer (NK) cells and M2 macrophages (Fig. 5A). Subsequently, we found that the expression of YTHDF2, an m6A ‘reader’ protein, was significantly lower in IgAN patients. In addition, the infiltration of M2 macrophages was lower in the IgAN group than that in the control group (Fig. 5B–D), indicating that the reduced M2 macrophage infiltration in IgAN may be closely related to the expression of YTHDF2.

**Fig. 6:**
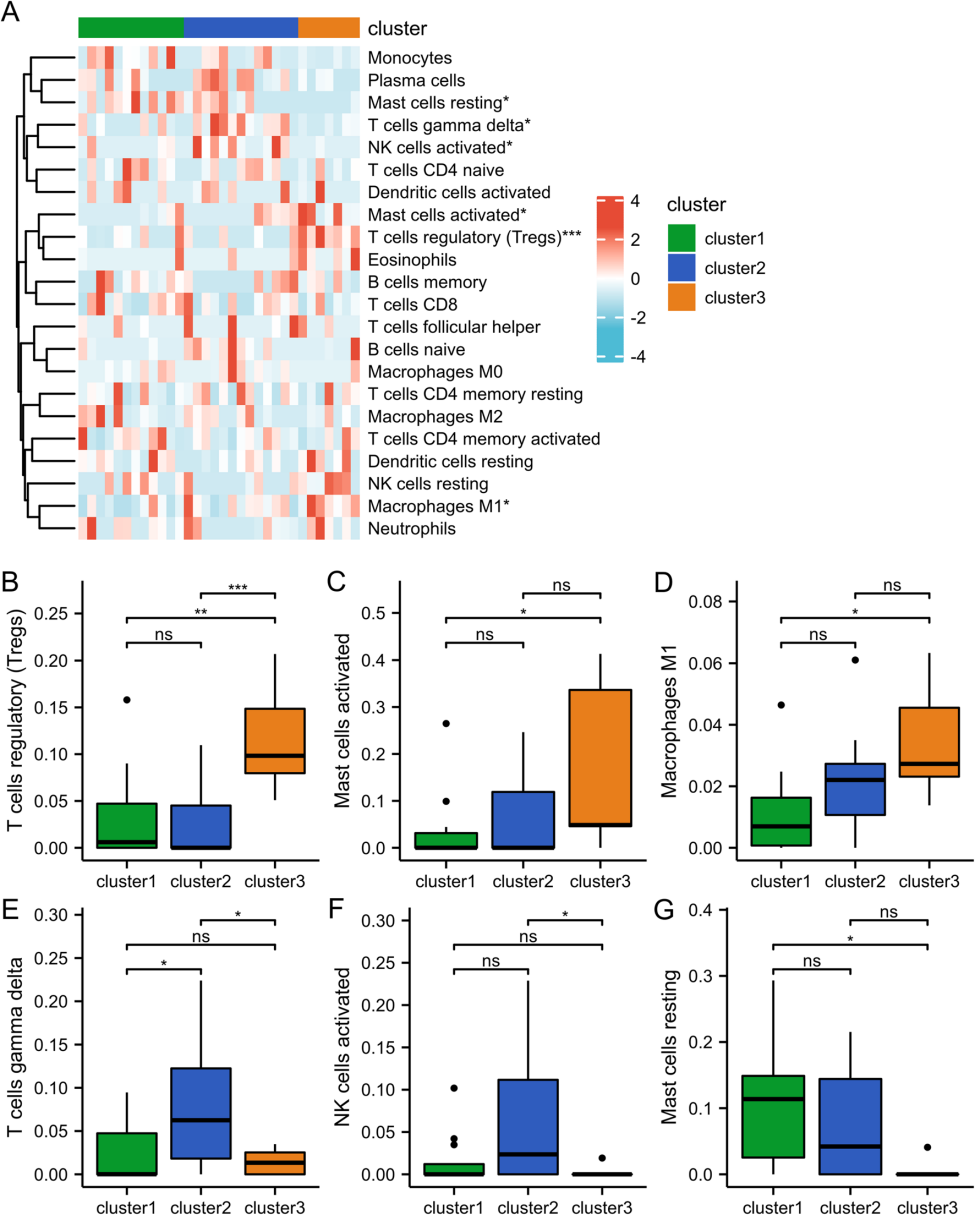
Differences in the level of immune cell infiltration among the 3 clusters. **A** The heatmap plot demonstrated the expression status of infiltrating immunocytes among the 3 m6A modification patterns. **B**–**D** the infiltrating levels of the T cells regulatory (**B**), mast cells activated (**C**), M1 macrophages (**D**), T cells gamma delta (**E**), NK cells activated (**F**), mast cells resting (**G**) in 3 clusters. *p < 0.05; **p < 0.01; ***p < 0.001

Infiltrating immunocytes were evaluated to understand the heterogeneity among immune microenvironment characteristics associated with different m6A modification clusters. In the comparison of the three clusters, 6 types of immunocytes were identified with different infiltration abundances (Fig. 6A). Compared with cluster-1 and cluster-2, the immunocyte infiltration of cluster-3 differed, featuring a higher level of regulatory T cells (Tregs), activated mast cells, and M1 macrophages and lower levels of gamma delta T cells, activated NK cells, and resting mast cells (Fig. 6B–G). These results show that m6A methylation modification plays an essential regulatory role in shaping different immune microenvironments in IgAN.

### Identification of m6A-related gene modules

**Fig. 7:**
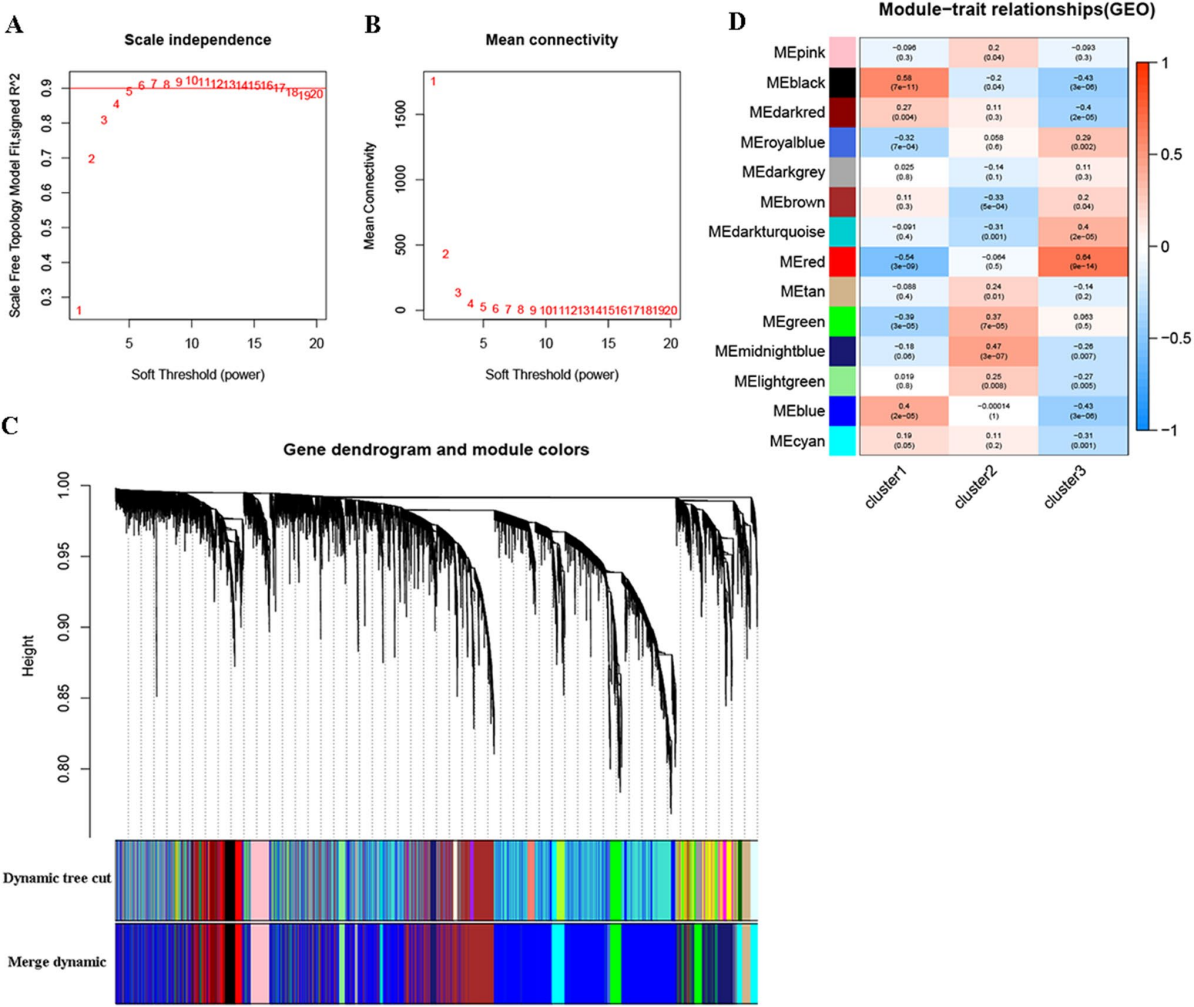
Identification and function analysis of m6A related genes in IgAN. **A** The *X*-axis reflects the soft-thresholding power. The *Y*-axis reflects the scale-free topology model fit index. The red line indicates *R*^2^ = 0.9. **B** The *X*-axis reflects the soft-thresholding power. The *Y*-axis reflects the mean connectivity. **C** Dendrogram of all genes clustered based on a dissimilarity measure (1-TOM). The color row underneath the dendrogram shows the module assignment determined by the Dynamic Tree Cut, in which 14 modules were identified. **D** Heatmap of the correlation between module genes and the m6A modification patterns

The soft-thresholding β power was calculated through network topology analysis and set to 5 because the scale independence reached 0.9 (Fig. 7A), displaying high average connectivity (Fig. 7B). Modules were generated by dynamic tree cutting and merged when the number of genes was less than 50, using cutting heights of 0.3 (Fig. 7C). Consequently, 14 modules were established for the whole transcriptome, and the gene modules with the highest correlations for each cluster were identified (Fig. 7D). The red module, containing 398 genes (Supplementary Table 1), showed the most significant positive correlation with cluster-3 (red module: *r* = 0.64, *p* < 0.001). These results laid the foundation for the study of gene expression mediated by m6A regulators.

### Validation of gene expression

**Fig. 8:**
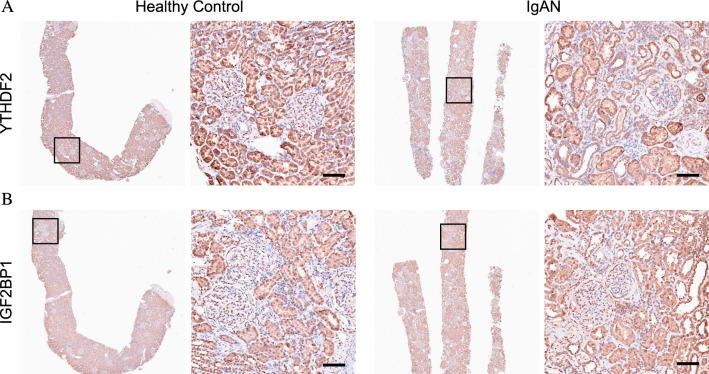
Representative images of YTHDF2 (**A**) and IGF2BP1 (**B**) staining of kidney biopsy from IgAN patients and healthy controls. Scale bar = 250 µm

Immunohistochemistry (IHC) analyses further confirmed that the protein levels of YTHDF2 were markedly decreased in IgAN patients compared with that in healthy controls. We observed that YTHDF2 were mainly expressed in the cytoplasm of renal tubular cells using IHC. YTHDF2 appeared to have a higher concentration in the cytoplasm of renal tubular cells from healthy controls rather than IgAN specimens. However, no difference in the expression of IGF2BP1 was observed between the two groups (Fig. 8). These results demonstrated that upregulated YTHDF2 may attenuate development of IgAN in vivo, indicating that YTHDF2 plays a protective role in IgAN. Thus, these findings indicate that YTHDF2 might act as a potential predictor in IgAN.

## Discussion

Although the pathogenesis of IgAN remains to be fully elucidated, episodic macroscopic hematuria concurrent with an infection of the upper respiratory tract suggests that the mucosal immune system plays an important role in the clinical manifestations of IgAN [15]. m6A modification plays critical roles in many regulatory processes, including pre-mRNA processing, mRNA export, mRNA stability, and translation efficiency. These m6A-mediated regulatory pathways affect many biological processes, such as development, the stress response, immune responses, and neuronal functions [16]. As our understanding of the importance of m6A modifications during physiological and pathological processes increases, fundamental roles for m6A in a variety of tumors continue to be identified [17]. In inflammatory diseases such as sepsis and periodontitis, changes in the expression patterns of m6A regulators affect the immune microenvironment and promote the occurrence of disease [18, 19]. Therefore, we further explored the role of m6A methylation in IgAN and immunocyte infiltration.

In this study, the expression patterns and immunocyte infiltration of m6A regulators in IgAN were explored. The biological functions of m6A are mediated through m6A reader proteins that specifically recognize and bind methylated adenosine bound to RNA, whereas the m6A modification is generated by m6A methylation writer proteins [20]. We constructed a model based on differences in the expression of m6A regulators to distinguish IgAN from other types of chronic nephritis. The good discrimination and calibration of the nomogram revealed that the m6A regulatory mechanism in IgAN was significantly different from that in the control group. A number of m6A regulators showed correlated expression patterns, revealing a regulatory network of m6A modifications. To explore the immune characteristics of the m6A regulatory network, correlations between m6A regulators and immunocyte infiltration in IgAN were investigated. The m6A regulator YTHDF2 was positively associated with the infiltration of M2 macrophages and negatively associated with the infiltration of resting NK cells. In a study of hepatocellular carcinoma, silenced YTHDF2 was found to provoke inflammation, vascular reconstruction, and metastatic progression [21]. Mechanistically, YTHDF2 inhibits the phosphorylation of signal transducer and activator of transcription 3 (STAT3) by degrading interleukin 11 (IL-11) mRNA, which encodes a dominant IL-6 family cytokine that endows gastrointestinal cancers with a proliferative and invasive capacity [22]. In animal models, YTHDF2 knockdown increased the mRNA expression of mitogen-activated protein kinase kinase 4 (MAP2K4) and mitogen-activated protein kinase kinase kinase kinase 4 (MAP4K4) by stabilizing mRNA transcripts, which activated mitogen-activated protein kinase (MAPK) and nuclear factor κB (NFκB) signaling pathways to promote macrophage activation [23]. In our study, we found that YTHDF2 was significantly decreased in IgAN samples. We suggest that YTHDF2 may function to protect parenchymal cells from excessive proinflammatory signals in IgAN. However, the precise role of YTHDF2 in IgAN remain further investigations. The classification strategy of consensus clustering for immune subtypes can help us understand the underlying mechanisms associated with immune regulation for the development of precise therapeutic strategies. Three different clusters based on differences in m6A expression were identified. Compared with the other two clusters, the expression of m6A in cluster-3 was generally lower. We observed that the infiltration abundance of Tregs in cluster-3 was significantly higher than those of the other two clusters. Previous studies have found that the number of Tregs in the peripheral blood of IgAN patients is significantly lower than that in healthy individuals [24]. After CD4^+^ CD25^+^ Tregs were amplified in vitro and infused into an IgAN rat model, urine protein levels and red blood cell counts improved, and glomerular injury was reduced based on pathological analysis [25]. Tregs are essential for the maintenance of immunological tolerance and immune homeostasis by suppressing the activation and expansion of potentially self-reactive T cells [26]. After treatment with CD4^+^ CD25^+^ Tregs, the expression of plasminogen activator inhibitor-1 (PAI-1), IL-6, and transforming growth factor-β1 (TGF-β1) mRNA and protein diminished significantly, reducing the proliferation of mesangial cells [27]. The theoretical basis of performing tonsillectomy to treat IgAN depends on increasing the level of CD4^+^ CD25^+^ Tregs [28]. These studies have shown that CD4^+^ CD25^+^ Tregs are closely related to the pathogenesis and disease progression of IgAN.

The heterogeneity of biological functions across different clusters helps to clarify the pathogenesis of IgAN from the perspective of m6A modifications. No significant differences in the activation status of hallmark pathways were observed between cluster-1 and cluster-2; therefore, we focused on the comparison between cluster-3 and the other two clusters. Compared with the other two clusters, the unfolded protein response (UPR) and DNA repair were significantly downregulated in cluster-3. Perturbations of kidney cells result in the accumulation of unfolded and misfolded proteins in the endoplasmic reticulum (ER), leading to the activation of the UPR or ER stress. The UPR might interact in a coordinated manner with autophagy to alleviate protein misfolding, which can lead to various kidney diseases, including diabetic nephropathy, acute kidney injury, chronic kidney disease and fibrosis [29, 30]. Proper regulation of the UPR might represent a promising therapeutic approach for preventing or arresting the progression of kidney disease [31]. Increased DNA damage has also been reported in acute kidney injury (AKI), drug-induced kidney injury, and various podocyte injuries [32–34]. These results suggest that activation of the DNA damage repair system is disease- and cell type specific. Although the repair capacity of different kidney cells has not been clearly identified, these findings present a new perspective from which to approach disease [35]. We also noticed that the KRAS signaling pathway, which was the only upregulated pathway identified in cluster-3, is the most frequently mutated oncogene in human cancers, with mutations identified in approximately 30% of all cancers [36]. The Ras protein family features three different isoforms (KRAS, HRAS, and NRAS) that are all expressed as membrane-bound proteins with inherent GTPase activity, activated by numerous extracellular stimuli and cycling between inactive (GDP-bound) and active (GTP-bound) forms. When bound to GTP, Ras proteins activate intracellular signaling pathways, which are critical for cell proliferation and angiogenesis [37]. Human mesangial cells require both KRAS and HRAS in vitro to undergo proliferation, and the downregulation of mesangial Ras in IgAN may, therefore, represent an adaptive response, limiting mesangial cell cycle progression [38]. We speculate that the IgAN disease severity associated with cluster-3 was milder than that associated with cluster-1 and -2, which would be consistent with the results of this immune characteristic. This classification strategy for IgAN subtypes can help us understand the underlying mechanisms associated with immune regulation and provide novel treatment perspectives for IgAN.

In this study, the relationship between m6A methylation regulators and IgAN was systematically analyzed, and m6A modifications were confirmed to be involved in the regulation of the immune microenvironment of IgAN. Our research also presents gene modules with the most significant correlations for each of the three clusters, laying the foundation for subsequent research to determine the key m6A regulators and immune characteristics associated with IgAN. However, this study also has some limitations that should be addressed. First, our conclusions are based on the bioinformatics analysis of datasets containing patient tissue, genetics, and other molecular information, and the findings here require further verification using biological experiments. Second, because this study was performed as an analysis of publicly available data, we were not able to obtain additional information regarding the demographics and clinical features of the patients, which could have provided more effective information regarding the pathogenesis and classification of IgAN. In addition, some datasets from the GEO database were not included in this study because they did not meet the selection criteria. The omission of some key clues might cause errors in the research conclusions.

## Conclusions

In summary, this research systematically evaluated the expression of m6A RNA regulators in IgAN and their correlations with the immune microenvironment. It was also identified that YTHDF2 was highly expressed in IgAN pathological sections. Through consensus clustering of m6A regulators, three IgAN clusters were identified, and differences in the levels of immunocyte infiltration among the clusters were determined. This comprehensive analysis confirmed the strong impact of m6A modifications on the immune characteristics of IgAN, inspiring new insights into more effective treatment methods.

## Supplementary Information

Below is the link to the electronic supplementary material.

Supplementary file1 (DOCX 94 kb)

## Data Availability

The datasets used and/or analyzed during the current study are available from NCBI Gene Expression Omnibus (GSE115857 and GSE116626, https://www.ncbi.nlm.nih.gov/geo/).
