# Supplementary material for: Molecular characterization of m6A RNA methylation regulators with features of immune dysregulation in IgA nephropathy
Source: Clin Exp Med. 2024 May 2;24(1):92. doi: 10.1007/s10238-024-01346-8 (PMC11062981; doi:10.1007/s10238-024-01346-8)
Supplement: Supplementary file 1 — Supplementary file1 (DOCX 94 kb) [file 10238_2024_1346_MOESM1_ESM.docx]

**Supplementary Figure 1:** The work flow of this study

**Supplementary table 1:** Gene list of red modules

CDC5L

CDH2

CDKN1C

CEBPD

CENTG3

CFB

CHADL

CHAF1B

CHCHD6

CHRNB1

CHST10

CHX10

CKB

CLCF1

CLIP3

CLPP

CLPX

CMTM8

CNGA1

CNTN6

COL28A1

COL6A6

CORO1C

COX11P

COX7B

COX8A

CPLX1

CPNE3

CPNE7

CPSF4L

CRIPT

CRKRS

CRLF2

CRNKL1

CSN3

CSPG5

CSTA

CTTNBP2

CUBN

CXCL10

CXXC4

CXorf22

CXorf41

CXorf57

CYBASC3

CYHR1

CYP21A2

CYP24A1

CYP26B1

CYP2E1

CYSLTR2

CYYR1

D2HGDH

D4S234E

DAAM2

DAXX

DDX10

DDX58

DES

DFNB31

DGAT1

DGKI

DGKQ

DISP1

DKFZp564N2472

DKFZp686D0972

DNAI1

DNAJB13

DNPEP

DPCR1

DPP9

DPY19L2P1

DPY19L4

DQX1

DRD4

DRG2

DSCR3

DSG1

DSG4

DUSP12

DUSP26

DVL1

DYSF

E2F5

EFHA1

EFHC1

EGF

EGLN1

EHF

ELAVL1

EMILIN3

EMP3

EPHA1

EPHB1

EPSTI1

ERAP2

EREG

EXOC3L

EXOC5

EXT1

EXTL1

F2

FADS3

FAM100B

FAM129A

FAM160A2

FAM180A

FAM195B

FAM3D

FAM80A

FAM83C

FAM83D

FAM90A7

FAM91A1

FCER2

FCHO2

FER1L6

FHOD3

FKBP5

FKBP6

FKSG29

FKSG30

FLJ16331

FLJ20628

FLJ25996

FLJ32011

FLJ33590

FLJ41603

FLJ43093

FLJ43276

FLJ44048

FLRT1

FMO5

GABRA2

GABRB1

GABRR2

GAGE5

GATA3

GDAP1L1

GDE1

GDI2

GHR

GIMAP5

GIPC1

GIPC2

GIPC3

GIYD2

GJB2

GJC3

GKAP1

GLOD4

GLT25D1

GNG2

GOLGA2LY2

GOLGA8A

GOLPH4

GPC2

GPHA2

GPR146

GPR4

GPR75

GPRC5A

GPSM2

GPX2

GRID2

GRIK5

GRN

GRTP1

GSTA5

GSX2

GTF3C4

GUCY1B3

GUCY2C

GUCY2D

H2AFZ

HBZ

HELB

HELZ

HHATL

HIST1H1A

HIST1H3C

HLA-DPB1

HMBOX1

HMGCL

HOOK1

HOOK3

HOXD10

HRG

HS2ST1

HS3ST3B1

HSH2D

HSP90AB1

HSPB8

HSPC171

HTR2B

IFIT1

IFNGR2

IFT172

IGFL3

IL6

IMPAD1

IMPDH2

INPP1

INPPL1

IQCE

IQGAP2

IRS1

ISG20L2

ISOC2

ISX

ITIH5L

ITPKB

IYD

JAGN1

KAZALD1

KCNE1

KCNJ12

KCNJ8

KHDRBS2

KIAA0087

KIAA1908

KIAA1919

KIAA2022

KIF2A

KIFC1

KIN

KLB

KLF13

KLHL10

LOC652276

LOC652968

LOC653545

LOC729905

LOC730862

LOC90586

LOC92017

LOR

LOX

LPCAT4

LPPR2

LRIG3

LRP2BP

LRRC18

LRRC33

LRRC8D

LXN

MAGOHB

MAP1LC3B

MAP3K8

MARCO

MATN1

MBD3

MBD3L5

MDGA1

MED29

METTL4

MFAP1

MFSD10

MFSD11

MGC10997

MGC21881

MICALL1

MICB

MIER2

MIP

MIR1296

MIR20B

MIR301A

MIR30B

MIR320B2

MIR490

MIR491

MIR548I1

MIR548K

MIR548L

MIR564

MIR567

MIR596

MIR597

MIR625

MIR626

MIR630

MIR643

MIR99B

MITD1

MKRN2

MMD

MMP24

MOCS3

MOGAT1

MON2

MORF4

MPZL3

MRGPRF

MRPS34

MRS2

MRS2P2

MS4A6E

MT4

MTDH

MTM1

MTMR1

MUC13

MUC15

MUCL1

MXD1

MYB

MYBL1

MYBPHL

MYD88

MYLC2PL

MYLK4

MYO1A

MYOZ1

NAPA

NAPB

NAPRT1

NBLA00301

NBPF20

NCRNA00093

NCRNA00113

NEIL2

NEK1

NFRKB

NFYB

NFYC

NKAIN3

NKG7

NOTCH4

NOVA2

NPAT

NPR3

NPY

NR1H4

NRARP

NRSN1

NT5M

NUDT16P

NUDT7

NUP210L

NXPH2

OBFC2B

OBSCN

ODC1

OPN1SW

OPTN

OR2A20P

OR51E1

OR52B6

OR7D2

ORC1L

OSTCL

OVOL2

P2RY4

P2RY5

PACSIN3

PAPOLB

PAQR8

PAR1

PCDHGA10

PCGF2

PCGF3

PCMTD2

PCOLCE2

PCSK5

PCSK7

PDCD1

PDE6B

PDIK1L

PDSS2

PEG3AS

PEX5

PEX7

PFKFB4

PGAM5

PGBD1

PGBD5

PGLS

PHCA

PHLPP2

PIK3R2

PISD

PLA2G4C

PREB

PRKAG3

PRPH2

PRRG3

RPS5

RRAGA

RS1

RSPH3

RSPH9

RSPO3

RXFP2

SAMD14

SATL1

SAV1

SBNO1
